# Association of gastroesophageal reflux disease with the incidence of multiple cancers: a systematic review and meta-analysis

**DOI:** 10.3389/fmed.2026.1765727

**Published:** 2026-02-26

**Authors:** XianHong Jiang, Xin Shao, Wenjie Zhou, Jie Dan, MingJie Zhu, Zhong Peng, Yong Hong Wang

**Affiliations:** 1 Department of Gastrointestinal Surgery, The People’s Hospital of Leshan, Leshan, China; 2 Department of Pharmacy, The People’s Hospital of Leshan, Leshan, China

**Keywords:** cancer, gastroesophageal reflux disease, meta-analysis, risk, tumor

## Abstract

**Objective:**

To investigate whether gastroesophageal reflux disease (GERD) is associated with an increased incidence of multiple cancers through a robust meta-analysis.

**Methods:**

We systematically searched PubMed, Embase, the Cochrane Library, and Web of Science for observational studies published up to July 11, 2025. All statistical analyses were performed using R version 4.5.0.

**Results:**

A total of 17 studies were included. The pooled results indicated that GERD was significantly associated with an increased risk of lung cancer (OR = 1.33, 95% CI: 1.25–1.42), laryngeal cancer (OR = 1.75, 95% CI: 1.38–2.21), pancreatic cancer (OR = 1.30, 95% CI: 1.12–1.50), and esophageal cancer (OR = 1.70, 95% CI: 1.12–2.57). However, no significant association was found between GERD and colorectal cancer (OR = 1.04, 95% CI: 0.63–1.72).

**Conclusion:**

This meta-analysis suggests that GERD is associated with an increased incidence of multiple cancers. These findings will contribute to the clinical management of GERD patients, particularly in terms of cancer prevention and early screening.

## Introduction

Gastroesophageal reflux disease (GERD) is a common digestive disorder characterized by the chronic backflow of stomach contents into the esophagus (1, 2), leading to symptoms such as heartburn and regurgitation. It is estimated that approximately 10–20% of the adult population in Western countries suffers from GERD, with increasing prevalence worldwide (3, 4). GERD is associated with significant morbidity and impaired quality of life. Long-term complications of GERD include esophageal erosions, peptic strictures, and Barrett’s esophagus, which can progress to esophageal adenocarcinoma (5). Additionally, GERD is a major cause of chronic cough, asthma, and dental erosion, further exacerbating the clinical burden of the disease (6).

Cancer remains one of the leading causes of morbidity and mortality worldwide, posing a substantial global public health burden. Emerging evidence suggests that gastroesophageal reflux disease (GERD) may contribute to the increased risk of several cancers. Chronic inflammation induced by prolonged acid reflux is believed to be a potential mechanism linking GERD to cancer development. For example, GERD has been strongly associated with an elevated risk of esophageal cancer, particularly esophageal adenocarcinoma (5, 7, 8). Additionally, some studies suggest that GERD may be linked to the incidence of lung cancer, laryngeal cancer, and other malignancies (9–12). However, the exact relationship remains controversial, with inconsistent findings across studies. For instance, some studies have shown that GERD does not increase the risk of esophageal cancer (13). Currently, no comprehensive meta-analysis has systematically evaluated the association between GERD and the incidence of multiple cancer types using cancer-specific analyses. Importantly, evaluating multiple cancer types within a unified analytical framework may help clarify whether GERD represents a broader cancer-related risk condition rather than an isolated risk factor for a single malignancy.

Therefore, this study aims to systematically evaluate the association between GERD and the incidence of lung cancer, laryngeal cancer, esophageal cancer, pancreatic cancer, and colorectal cancer based on available epidemiological observational evidence. The findings will contribute to the clinical management of GERD patients, particularly in terms of cancer prevention and early screening. By better understanding the risks associated with GERD, we hope to improve patient outcomes and guide clinical practices in identifying high-risk individuals for early interventions.

## Methods

This systematic review and meta-analysis was conducted in accordance with the Preferred Reporting Items for Systematic Reviews and Meta-Analyses (PRISMA) guidelines (14) and, where applicable, the Meta-analysis Of Observational Studies in Epidemiology (MOOSE) guidelines (15). This meta-analysis has been registered in PROSPERO (CRD420251122941).

### Search strategy

We conducted a literature search across multiple databases, including PubMed, Embase, the Cochrane Library, and Web of Science, targeting observational studies published before July 11, 2025. The search strategy utilized key terms such as “Gastroesophageal Reflux,” “gastroesophageal reflux disease,” “Neoplasms,” “Cancer,” and “Cohort Studies.” Details of the full search methodology are provided in Supplementary Table S1. To enhance the comprehensiveness of our search, we also reviewed the reference lists of all included articles.

### Eligibility criteria

Criteria for inclusion: (1) GERD was diagnosed based on typical symptoms such as acid reflux and heartburn, and confirmed through gastroscopy, proton pump inhibitor (PPI) testing, or 24-h esophageal pH monitoring in both adults and children; (2) cancer diagnoses were confirmed by imaging techniques, International Classification of Diseases (ICD) codes, histopathological examination, or reliable medical records; (3) studies comparing the incidence of various cancers between participants with and without GERD, with results reported as odds ratios (ORs) and corresponding 95% confidence intervals (CIs), or providing sufficient data to calculate these estimates; (4) full-text articles were available for review.

Criteria for exclusion: (1) meta-analyses, practice guidelines, conference abstracts, animal studies, commentaries, reviews, case–control studies, or case reports; (2) studies not available in full text; (3) duplicate studies; (4) lack of relevant data or no outcome of interest.

### Study selection

The literature search was independently conducted by two researchers (XJ and XS). After duplicates were removed, studies irrelevant to the topic were excluded based on title and abstract screening. The full texts of the remaining studies were then retrieved and carefully evaluated for inclusion eligibility. In cases of disagreement during the selection process, a third researcher was consulted to reach a final consensus.

### Data extraction and outcome measures

A pre-designed table was used to collect relevant data, including the first author’s name, country, year of publication, population characteristics, sample size, follow-up time, confirmation of GERD and cancer, and reported endpoints of interest. All of these steps were independently performed by two individuals (XJ and XS), with any discrepancies resolved through discussion.

### Risk of bias assessment

All the studies included in our meta-analysis were cohort studies. Therefore, two reviewers (XJ and XS) independently assessed the risk of bias in each included study using The Newcastle-Ottawa Scale (NOS) (16). This scale assesses the quality of studies in three domains: selection, comparability, and outcome. In the selection domain, a study may earn as many as four stars; in comparability, up to two stars; and in outcome, a maximum of three stars. Overall, the total scores for studies range from zero to nine.

### Statistical analysis

To assess heterogeneity across the included studies, the *I*^2^ statistic and Cochran’s *Q* test were employed. When substantial heterogeneity was observed (*I*^2^ > 50% and/or *p* < 0.1), a random-effects model was applied; otherwise, a fixed-effects model was used. Sensitivity analyses were conducted to explore potential sources of heterogeneity and evaluate the robustness of the pooled estimates. Publication bias in the association between GERD and various types of cancer was examined using funnel plots, together with Egger and Begg tests. All statistical analyses and visualizations were performed using R software (version 4.5.0).

## Results

### Literature search

**Figure 1:**
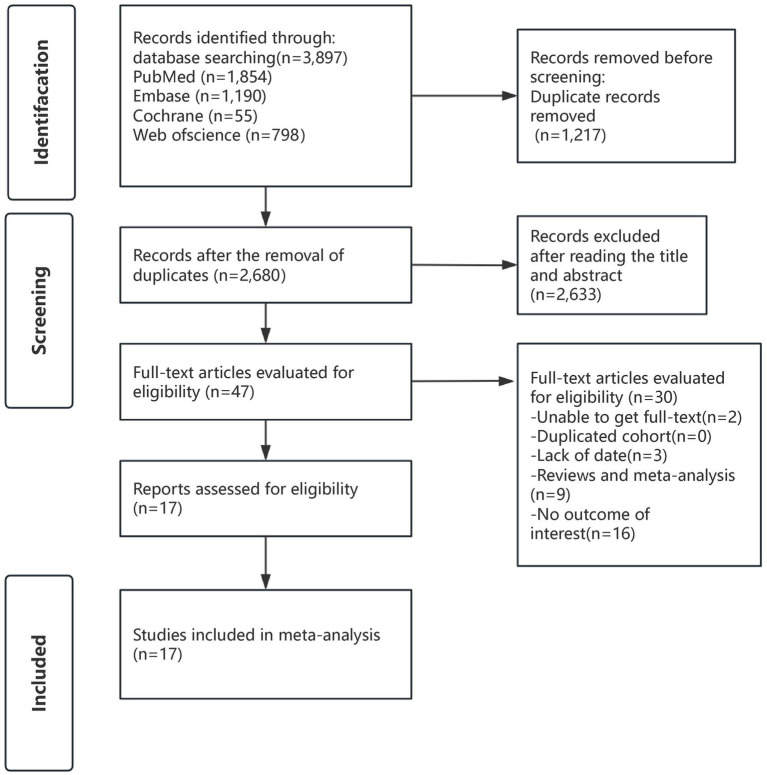
Search strategy diagram.

A comprehensive search was conducted for studies published before July 11, 2025, resulting in 3,897 records. After removing duplicates, 2,680 records were left. Initial screening based on titles and abstracts led to the exclusion of 2,633 records. The full texts of the remaining 47 articles were reviewed in depth, with 30 studies being excluded for reasons outlined in Supplementary Table S2. In the end, 17 studies were included in this meta-analysis. The screening process is shown in Figure 1.

### Study characteristics

The basic characteristics of the 17 included studies (7, 12, 17–31) are detailed in Table 1. These studies were published between 2016 and 2025 and were conducted in various countries, including three studies from China (18, 24, 27), six studies from the United States (17, 19, 20, 23, 25, 26), three studies from South Korea (21, 22, 29), 1 study from Iran (28), and four studies from the United Kingdom (7, 12, 30, 31). The studies involved a total of 4,049,027 participants. Regarding cancer types, lung cancer was investigated in six studies (7, 18, 21, 26, 27, 30), while laryngeal cancer was examined in seven studies (17–20, 22, 23, 25). Esophageal cancer was assessed in five studies (7, 25, 28, 29, 31), and colorectal cancer was addressed in three studies (7, 24, 29). Pancreatic cancer was explored in three studies (7, 12, 29), and thyroid cancer was examined in one study (29).

**Table 1:** Basic characteristics of included studies.

Year	Country	Author	Sample size	Follow-up time (mean years)	Number of GERD cases	Male (%)	Age (years)	Diagnosis of MASLD	Ascertain of cancers	Endpoints	NOS scores
2016	China	Hsu et al. (18)	76,369	6	15,412	48.86	52 means	ICD codes	ICD codes	Lung cancer	8
2021	China	Hu et al. (24)	274,968	10	45,828	48.4	46 means	ICD codes	ICD codes	Colorectal cancer	8
2023	China	Li et al. (27)	602,604	NR	129,080	NR	NR	ICD codes	ICD codes	Lung cancer	8
2023	Iran	Soroush et al. (28)	49,559	13	9,005	42.4	50 means	ICD codes	ICD codes	Esophageal cancer	8
2019	South Korea	Choi et al. (21)	1,070	6.5	427	47.5	65 means	ICD codes	ICD codes	Lung cancer	6
2019	South Korea	Kim et al. (22)	296,121	11	98,707	45.7	58 means	ICD codes	ICD codes	Laryngeal cancer	8
2023	South Korea	Tran et al. (29)	514,866	9.9	10,872	50.92	54 means	ICD codes	ICD codes	Esophageal cancer, laryngeal cancer, thyroid cancer, colorectal cancer, liver cancer, pancreatic cancer	9
2012	The United Kingdom	Macfarlane et al. (31)	3,761	NR	1,789	NR	NR	ICD codes	ICD codes	Esophageal cancer	7
2024	The United Kingdom	Liao et al. (30)	501,569	11.54	58,191	45.58	40-69	ICD codes	ICD codes	Lung cancer	8
2024	The United Kingdom	Wu et al. (7)	602,604	NR	129,080	NR	NR	ICD codes	ICD codes	Lung cancer, esophageal cancer, pancreatic cancer, colorectal cancer	9
2025	The United Kingdom	Yang et al. (12)	602,604	NR	129,080	NR	NR	ICD codes	ICD codes	Pancreatic cancer	8
2016	The United States	Busch et al. (17)	2,571	NR	569	77.1	66 means	ICD codes	ICD codes	Laryngeal cancer	6
2018	The United States	Riley et al. (20)	27,610	NR	6,946	77.94	66-99	ICD codes	ICD codes	Laryngeal cancer	8
2018	The United States	Anis et al. (19)	2,730	6.5	413	47.7	69 means	ICD codes	ICD codes	Laryngeal cancer	7
2020	The United States	Parsel et al. (23)	2,094	NR	478	75.4	69 means	ICD codes	ICD codes	Laryngeal cancer	6
2021	The United States	Wang et al. (25)	490,605	15.5	116,476	NR	50-71	Medical records	ICD codes	Laryngeal cancer, esophageal cancer	7
2022	The United States	Amarnath et al. (26)	1,083	NR	174	25.7	72 means	ICD codes	ICD codes	Lung cancer	6

### Assessment of quality of included studies

The 17 studies included in this systematic review consisted of 13 studies of high quality, with scores ranging from 7 to 9 according to the NOS criteria. Four studies scored 6, indicating moderate quality. The specific scores for each study are provided in Table 1.

### Risk of lung cancer

**Figure 2:**
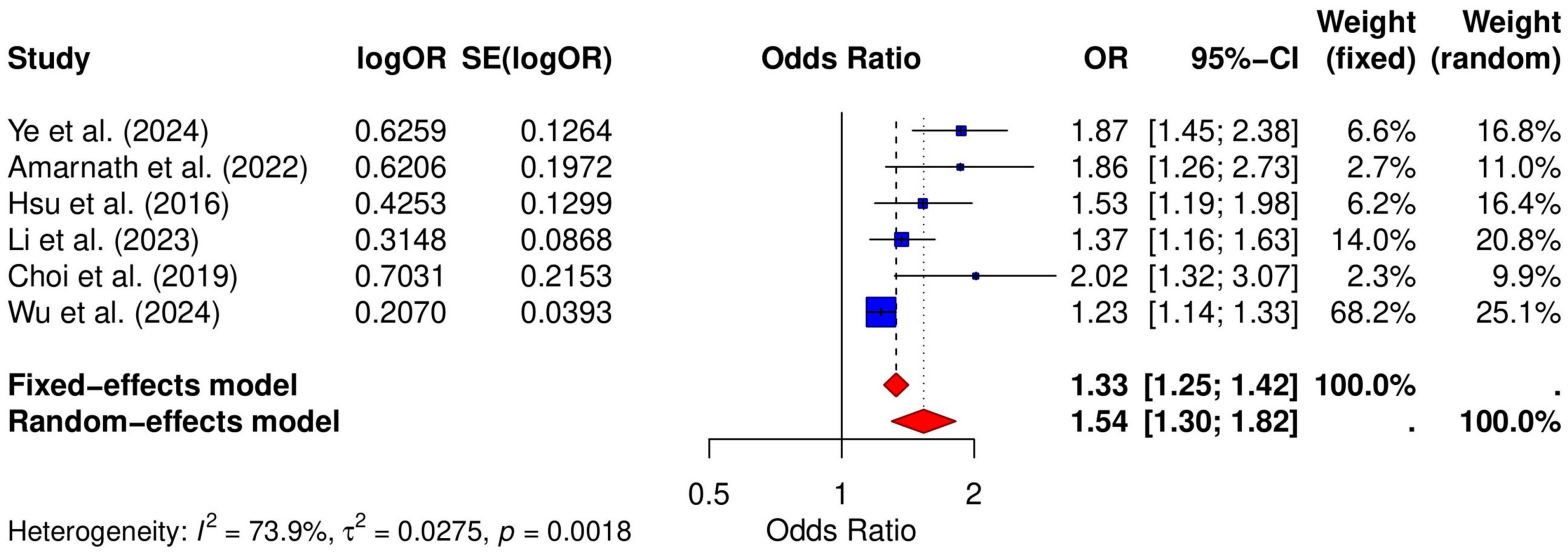
Forest plot of the GERD and the risk of lung cancer.

Six studies analyzed the relative risk of lung cancer in GERD patients. The pooled analysis revealed a significant positive association between GERD and lung cancer risk (OR = 1.33, 95% CI: 1.25–1.42, *p* = 0.0018; *I*^2^ = 73.9%; Figure 2). Publication bias was assessed using both the Begg and Egger tests. The Begg test showed no significant bias (*p* = 0.0909), while the Egger test indicated some degree of publication bias (*p* = 0.0042). Funnel plot analysis also suggested some asymmetry (Supplementary Figure S1), further supporting the possibility of publication bias. Sensitivity analysis demonstrated that the pooled OR remained significant after excluding individual studies, indicating the robustness of the results (Supplementary Figure S2).

### Risk of laryngeal cancer

**Figure 3:**
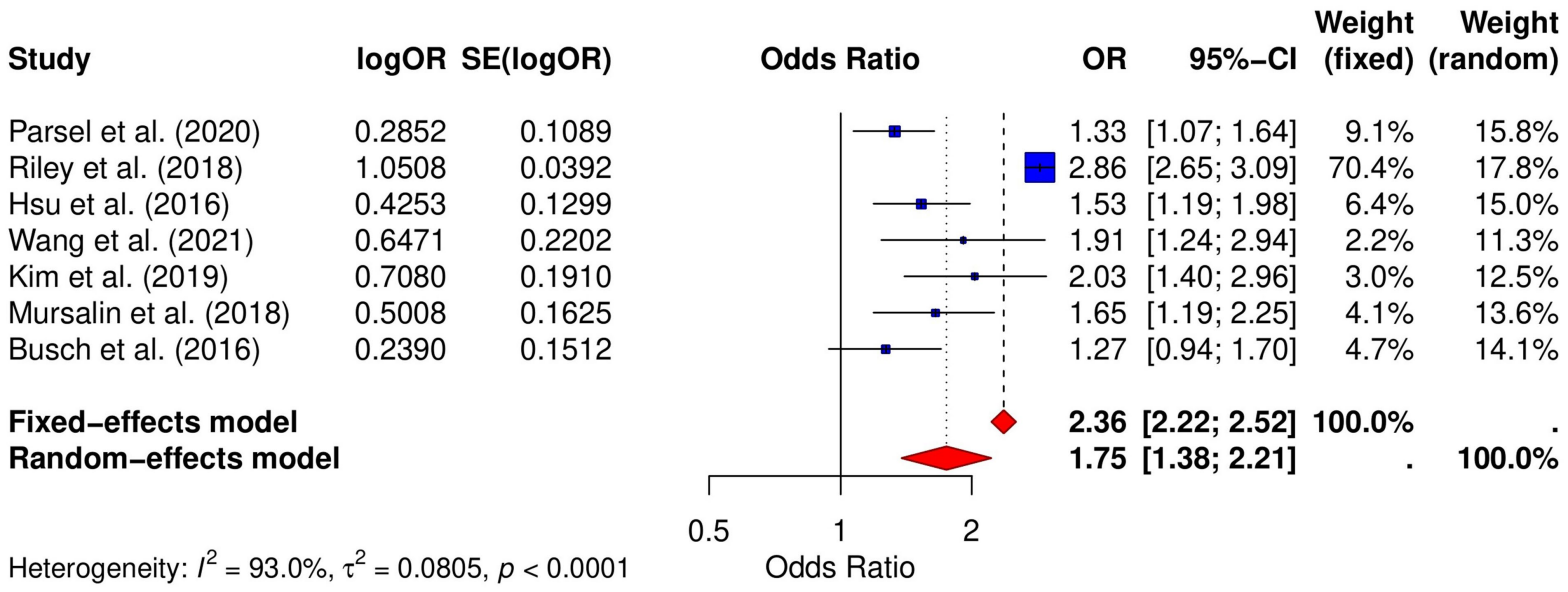
Forest plot of the GERD and the risk of laryngeal cancer.

Seven studies analyzed the relative risk of laryngeal cancer in GERD patients. The results showed that the risk of laryngeal cancer in GERD patients was significantly increased (OR = 1.75, 95% CI: 1.38–2.21, *p* < 0.000118; *I*^2^ = 93.0%; Figure 3). Publication bias was assessed using both the Begg and Egger tests. The Begg test showed no significant bias (*p* = 0.4527), while the Egger test indicated some degree of publication bias (*p* = 0.0237). Funnel plot analysis also suggested some asymmetry (Supplementary Figure S3), which may further support the possibility of publication bias. Sensitivity analysis demonstrated that the pooled OR remained significant after omitting individual studies, suggesting the robustness of the results (Supplementary Figure S4).

### Risk of esophageal cancer

**Figure 4:**
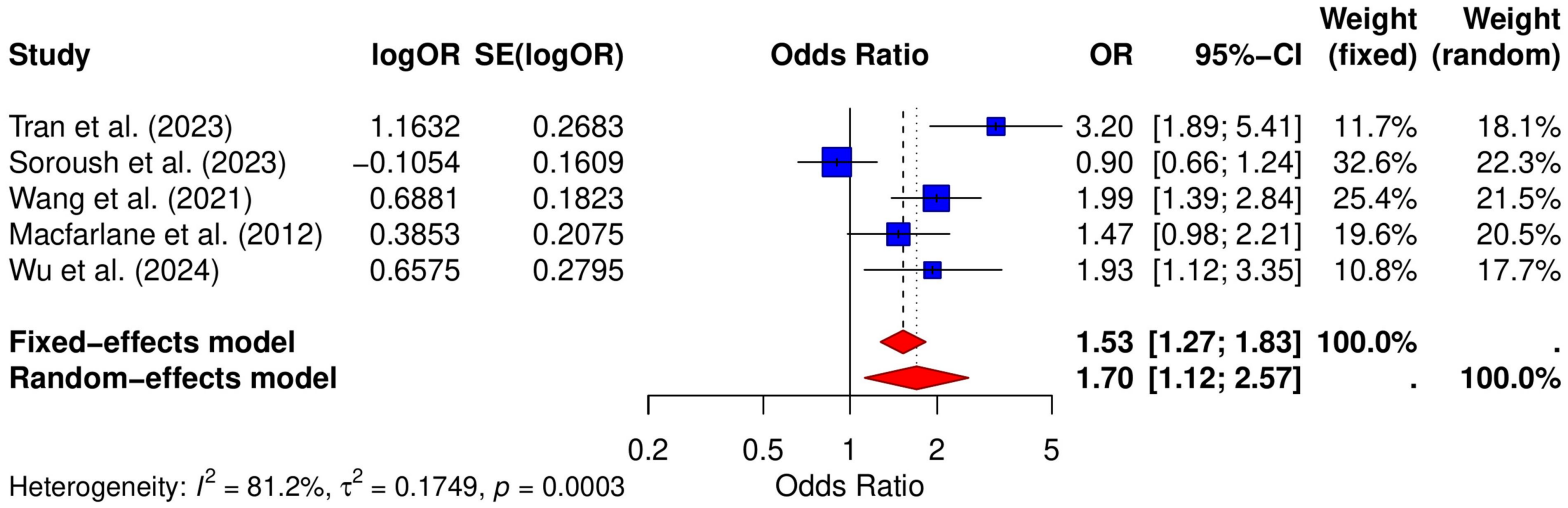
Forest plot of the GERD and the risk of esophageal cancer.

Five studies analyzed the relative risk of esophageal cancer in GERD patients. The meta-analysis in this study showed a significant increase in the risk of esophageal cancer in GERD patients (OR = 1.70, 95% CI: 1.12–2.57, *p* = 0.0003; *I*^2^ = 81.2%; Figure 4). Publication bias was assessed using both the Begg and Egger tests. The Begg test showed no significant bias (*p* = 0.3272), and the Egger test also showed no significant bias (*p* = 0.1496). Funnel plot analysis demonstrated symmetry, further confirming the absence of substantial publication bias (Supplementary Figure S5). Sensitivity analysis indicated that the pooled OR remained significant after omitting individual studies, suggesting the robustness of the results (Supplementary Figure S6).

### Risk of pancreatic cancer

**Figure 5:**
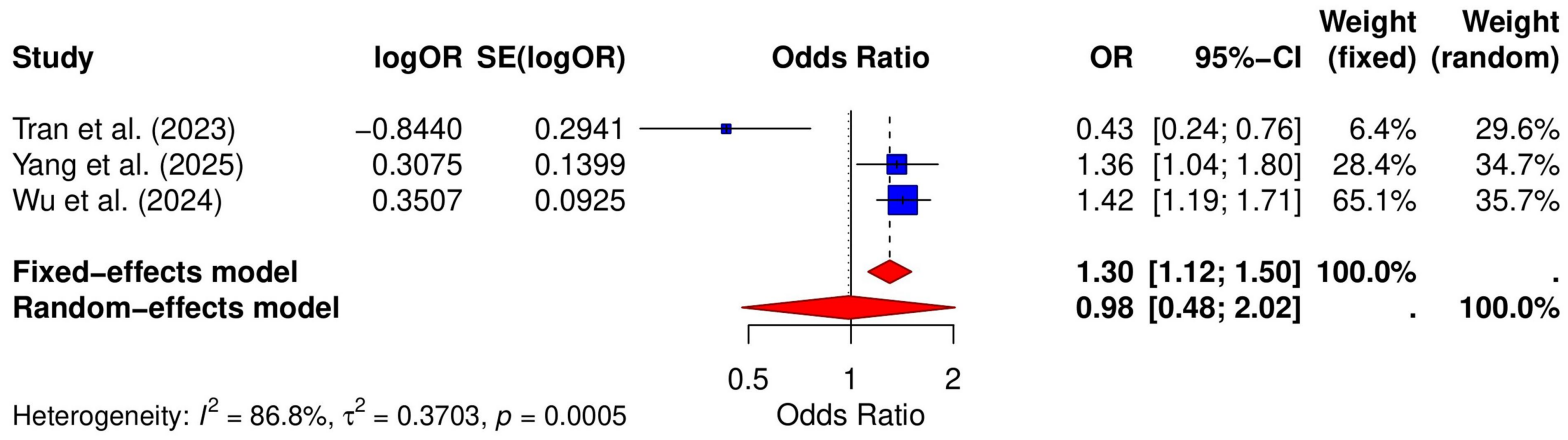
Forest plot of the GERD and the risk of pancreatic cancer.

Three studies assessed the relative risk of pancreatic cancer in GERD patients. The meta-analysis revealed a pooled OR of 1.30 (95% CI: [1.12; 1.50]) from the fixed-effect model, and the pooled OR from the random-effects model was 0.98 (95% CI: [0.48; 2.02]) with high heterogeneity (*I*^2^ = 86.8%) (Figure 5). Publication bias was evaluated using both Begg and Egger tests, with Begg’s *p*-value of 0.1172 and Egger’s *p*-value of 0.2322, indicating no significant bias. Funnel plot analysis confirmed symmetry, further supporting the absence of substantial publication bias (Supplementary Figure S7). Sensitivity analysis showed that the pooled OR remained significant after excluding individual studies, indicating the robustness of the results (Supplementary Figure S8).

### Risk of colorectal cancer

**Figure 6:**
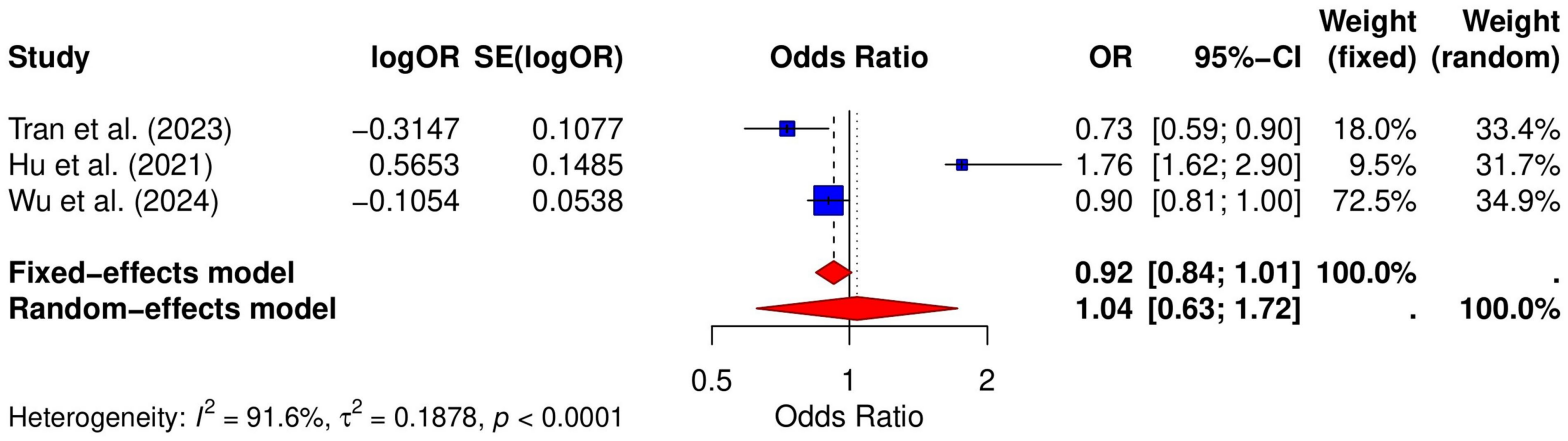
Forest plot of the GERD and the risk of colorectal cancer.

Three studies analyzed the relative risk of colorectal cancer in GERD patients. The results showed no significant positive association between GERD and colorectal cancer risk (OR = 1.04, 95% CI: 0.63–1.72, *p* < 0.0001; *I*^2^ = 91.6%; Figure 6). Publication bias was assessed using both the Begg and Egger tests. The Begg test showed no significant bias (*p* = 0.6015), and the Egger test also showed no significant bias (*p* = 0.6739). Funnel plot analysis demonstrated symmetry (Supplementary Figure S9), further confirming the absence of substantial publication bias. Sensitivity analysis indicated that the pooled OR remained significant after excluding individual studies, suggesting the robustness of the results (Supplementary Figure S10).

## Discussion

In this study, we performed a meta-analysis to evaluate the association between GERD and various cancers, including lung cancer, laryngeal cancer, esophageal cancer, pancreatic cancer, and colorectal cancer. The results provided valuable insights into the potential risks GERD may pose in relation to these cancers.

For lung cancer, our study found that GERD significantly increases the incidence of lung cancer, with a 30% higher risk. This finding is consistent with previous studies (11), which have similarly shown an association between GERD and an elevated risk of lung cancer. The potential mechanism underlying this relationship may be chronic inflammation caused by prolonged acid reflux, which could promote carcinogenesis in the lung tissue (32). Additionally, GERD-related risk factors, such as smoking and aspiration-related airway inflammation, might further exacerbate the risk of lung cancer development (33). Repeated microaspiration of refluxate (acid, bile acids, and pepsin) into the airway may further aggravate bronchial epithelial injury and promote a pro-inflammatory microenvironment.

For laryngeal cancer, prior studies have demonstrated an increased incidence of laryngeal cancer in GERD patients (34–37), and our findings also support this association. Chronic acid reflux can lead to irritation and inflammation of the laryngeal mucosa, increasing the likelihood of malignant transformation (38, 39). This is consistent with the concept of laryngopharyngeal reflux, in which refluxate reaches the larynx and pharynx and causes long-term mucosal inflammation. This mechanism may contribute to the elevated risk of laryngeal cancer in GERD patients. Given the high heterogeneity observed in the studies, further research is needed to explore additional factors that may influence this association.

Regarding esophageal cancer, there has been ongoing debate in the literature, with some studies suggesting an increased risk (8) and others showing no effect (13). Our meta-analysis provides evidence for a significant association between GERD and esophageal cancer. The potential mechanism may involve the development of Barrett’s esophagus, a condition that is strongly linked to GERD and significantly increases the risk of esophageal adenocarcinoma (40–42). However, the high heterogeneity observed in the included studies indicates that factors such as the severity of reflux and the presence of Barrett’s esophagus may contribute to the variability in results.

Regarding pancreatic cancer, the pooled OR from the fixed-effect model was 1.30 (95% CI: [1.12; 1.50]), suggesting a potential increase in risk. However, the pooled OR from the random-effects model was 0.98 (95% CI: [0.48; 2.02]), indicating no significant increase in risk, possibly due to heterogeneity between studies (*I*^2^ = 86.8%). The inconsistency between the two models may be influenced by differences in study design, sample size, or population characteristics. Although the underlying mechanism remains unclear, GERD-related systemic inflammation and metabolic risk factors (e.g., obesity) may contribute; however, current evidence remains insufficient to draw firm conclusions. Future research with larger and more homogeneous cohorts is necessary to further clarify the potential link between GERD and pancreatic cancer.

For colorectal cancer, our results showed no significant association between GERD and colorectal cancer incidence. However, the limited number of studies included in our analysis restricts the reliability of this conclusion, and further research is needed to clarify the relationship. Interestingly, some studies have suggested that GERD patients may have an increased risk of developing colorectal polyps (43). This raises the possibility that GERD-related factors, such as chronic inflammation or alterations in gut microbiota due to acid reflux, might contribute to the development of precancerous lesions in the colon. Given the potential implications, more comprehensive studies are warranted to explore whether GERD plays a role in colorectal cancer risk and to identify underlying mechanisms.

To our knowledge, this is the most comprehensive and up-to-date meta-analysis evaluating the relationship between GERD and the risk of various cancers, including lung cancer, esophageal cancer, pancreatic cancer, and colorectal cancer. This study integrates data from multiple studies, providing a robust estimate of the association between GERD and these cancer risks across different populations. By using rigorous statistical methods such as fixed-effect and random-effects models, publication bias assessments, and sensitivity analysis, we have ensured the reliability and validity of our findings.

However, several limitations must be acknowledged. First, the high heterogeneity observed in some of the analyses, particularly for pancreatic and colorectal cancers, suggests that variability across studies may affect the generalizability of the results. This could be attributed to differences in study design, sample sizes, and population characteristics. In addition, variations in follow-up duration across included cohort studies may have contributed to heterogeneity in the pooled estimates. Second, while publication bias was assessed using both Begg and Egger tests, the presence of publication bias in some analyses, particularly in lung cancer, could potentially influence the overall conclusions. Third, the limited number of studies available for certain cancer types, such as colorectal cancer, restricts the strength of the conclusions drawn for those specific cancers. In addition, the insufficient number of included studies precluded further subgroup analyses based on factors such as age, sex, geographic region, or GERD severity, or study quality (NOS score), which may have provided more detailed insights into potential sources of heterogeneity. Finally, the observational nature of the included studies means that causality cannot be definitively established, and residual confounding factors may still influence the observed associations.

## Conclusion

In conclusion, this study aimed to explore the association between GERD and the incidence of various cancers. We found that gastroesophageal reflux disease (GERD) is associated with an increased incidence of lung cancer, laryngeal cancer, esophageal cancer, and pancreatic cancer, while no significant association was found with the incidence of colorectal cancer. The findings of this study will contribute to the clinical management of patients with GERD. Future well-designed prospective studies with larger sample sizes are warranted to further clarify the causal relationship between GERD and cancer risk and to explore potential effect modifiers through detailed subgroup analyses.

## Data Availability

The original contributions presented in the study are included in the article/Supplementary material, further inquiries can be directed to the corresponding author.
