# Supplementary material for: Association of gastroesophageal reflux disease with the incidence of multiple cancers: a systematic review and meta-analysis
Source: Front Med (Lausanne). 2026 Feb 26;13:1765727. doi: 10.3389/fmed.2026.1765727 (PMC12980549; doi:10.3389/fmed.2026.1765727)
Supplement: Supplementary file 1 [file Supplementary_file_1.docx]

| **Table S1.** **details of search strategy (from database inception to July 11, 2025)** | | |  |  |
| --- | --- | --- | --- | --- |
| **No.** | **Search Strategies** | **Results** |  |  |
| **PubMed** | | |  |  |
| #1 | "Gastroesophageal Reflux"[Mesh] | 30824 |  |  |
| #2 | "Gastroesophageal Reflux"[Mesh] OR "gastroesophageal reflux disease"[Title/Abstract] OR "acid reflux"[Title/Abstract] OR "reflux esophagitis"[Title/Abstract] OR "GERD"[Title/Abstract] OR "heartburn"[Title/Abstract] OR "reflux disease"[Title/Abstract] | 44,523 |  |  |
| #3 | "Neoplasms"[Mesh] | 4,139,638 |  |  |
| #4 | ((("Neoplasms"[Mesh]) OR ("Cancer"[Title/Abstract])) OR ("Malignancy"[Title/Abstract])) OR ("Carcinoma"[Title/Abstract]) OR ("Tumor"[Title/Abstract]) | 5,327,448 |  |  |
| #5 | #2 AND #4 | 6934 |  |  |
| #6 | ((("Cohort Studies"[Mesh]) OR (cohort[Title/Abstract])) OR ("case-control studies"[Mesh]) OR ("observational study"[Title/Abstract])) | 3,518,657 |  |  |
| #7 | #5 AND #6 | 1,854 |  |  |
| **Embase** | | |  |  |
| #1 | ‘Gastroesophageal Reflux’/exp | 85,332 |  |  |
| #2 | 'gastroesophageal reflux':ab,ti OR 'gastroesophageal reflux disease':ab,ti OR 'acid reflux':ab,ti OR 'reflux esophagitis':ab,ti OR 'GERD':ab,ti OR 'heartburn':ab,ti OR 'reflux disease':ab,ti | 64,343 |  |  |
| #3 | ‘Neoplasms’/exp | 331,912 |  |  |
| #4 | 'neoplasms':ab,ti OR 'cancer':ab,ti OR 'malignancy':ab,ti OR 'carcinoma':ab,ti OR 'tumor':ab,ti | 5,346,715 |  |  |
| #5 | (#1 OR #2) AND (#3 OR #4) | 10,790 |  |  |
| #6 | ‘Cohort studies’/exp | 489,116 |  |  |
| #7 | 'cohort':ab,ti OR 'case-control':ab,ti OR 'observational study':ab,ti | 2,076,904 |  |  |
| #8 | #5 AND (#6 OR #7) | 1,190 |  |  |
| **Cochrane Library** | | |  |  |
| #1 | MeSH descriptor: [Gastroesophageal Reflux] explode all trees | 5,346 |  |  |
| #2 | ("gastroesophageal reflux"):ab,ti,kw OR ("gastroesophageal reflux disease"):ab,ti,kw OR ("acid reflux"):ab,ti,kw OR ("reflux esophagitis"):ab,ti,kw OR ("GERD"):ab,ti,kw OR ("heartburn"):ab,ti,kw OR ("reflux disease"):ab,ti,kw | 8,181 |  |  |
| #3 | MeSH descriptor: [Neoplasms] explode all trees | 111,089 |  |  |
| #4 | ("neoplasms"):ab,ti,kw OR ("cancer"):ab,ti,kw OR ("malignancy"):ab,ti,kw OR ("carcinoma"):ab,ti,kw OR ("tumor"):ab,ti,kw | 280,631 |  |  |
| #5 | MeSH descriptor: [Cohort Studies] explode all trees | 40,315 |  |  |
| #6 | ("cohort"):ab,ti,kw OR ("case-control"):ab,ti,kw OR ("observational study"):ab,ti,kw | 102,215 |  |  |
| #7 | (#1 OR #2) AND (#3 OR #4) AND (#5 OR #6) | 55 |  |  |
| **Web of science** | | |  | 59 |
| #1 | TS=("gastroesophageal reflux" OR "gastroesophageal reflux disease" OR "acid reflux" OR "reflux esophagitis" OR "GERD" OR "heartburn" OR "reflux disease") | 48,323 |  |  |
| #2 | TS=("neoplasms" OR "cancer" OR "malignancy" OR "carcinoma" OR "tumor") | 4,646,868 |  |  |
| #3 | #1 AND #2 | 5,802 |  |  |
| #4 | TS=("Cohort Studies" OR "cohort" OR "case-control" OR "case control" OR "observational study") | 1,388,831 |  |  |
| #5 | #3 AND #4 | 798 |  |  |

| **Table S2. Reason description for exclusion of ?? studies** | | | |
| --- | --- | --- | --- |
| **No.** | **First author** | **Publication year** | **Reason for exclusion** |
| 1 | Rune Erichsen | 2012 | Lack of relevant data |
| 2 | Evan L Busch | 2016 | Lack of relevant data |
| 3 | Monica E D'Arcy | 2025 | Lack of relevant data |
| 4 | Duo Zhan | 2014 | Meta-analyses |
| 5 | Michael B Cook | 2014 | Meta-analyses |
| 6 | Sean M Parsel | 2019 | Meta-analyses |
| 7 | Annica C Eells | 2020 | Meta-analyses |
| 8 | Bibek Saha | 2024 | Meta-analyses |
| 9 | Xin Wang | 2025 | Meta-analyses |
| 10 | Jalal Rezaii | 2008 | No outcome of interest |
| 11 | Nitin Shivappa | 2015 | No outcome of interest |
| 12 | Amnon Sonnenber | 2018 | No outcome of interest |
| 13 | Shyam Menon | 2019 | No outcome of interest |
| 14 | Manar Yanes | 2020 | No outcome of interest |
| 15 | Xiaotao Zhang | 2021 | No outcome of interest |
| 16 | Amin Andalib | 2021 | No outcome of interest |
| 17 | Tereza Deissova | 2022 | No outcome of interest |
| 18 | Manar Yanes | 2022 | No outcome of interest |
| 19 | Dag Holmberg | 2022 | No outcome of interest |
| 20 | Xuening Zhang | 2023 | No outcome of interest |
| 21 | Johan Hardvik Åkerström | 2024 | No outcome of interest |
| 22 | Apoorva Krishna Chandar | 2024 | No outcome of interest |
| 23 | Christopher J Byrne | 2024 | No outcome of interest |
| 24 | Peter K M Ku | 2024 | No outcome of interest |
| 25 | Tz-Wei Chiou | 2025 | No outcome of interest |
| 26 | J Veziant | 2023 | Reviews |
| 27 | Shuangyue Wang | 2024 | Reviews |
| 28 | Ahmed Edhi | 2024 | Reviews |
| 29 | Scott M Langevin | 2013 | Unable to get full-text |
| 30 | Maja Sereg-Bahar | 2015 | Unable to get full-text |
| **Excluded studies for the table[1-30]**  1. Erichsen, R., et al., *Erosive reflux disease increases risk for esophageal adenocarcinoma, compared with nonerosive reflux.* Clin Gastroenterol Hepatol, 2012. **10**(5): p. 475-80.e1.  2. Busch, E.L., J.P. Zevallos, and A.F. Olshan, *Gastroesophageal reflux disease and odds of head and neck squamous cell carcinoma in North Carolina.* Laryngoscope, 2016. **126**(5): p. 1091-6.  3. D'Arcy, M.E., et al., *Inflammatory diseases and risk of lung cancer among individuals who have never smoked.* Nat Commun, 2025. **16**(1): p. 5095.  4. Zhang, D., et al., *Gastroesophageal reflux and carcinoma of larynx or pharynx: a meta-analysis.* Acta Otolaryngol, 2014. **134**(10): p. 982-9.  5. Cook, M.B., et al., *Gastroesophageal reflux in relation to adenocarcinomas of the esophagus: a pooled analysis from the Barrett's and Esophageal Adenocarcinoma Consortium (BEACON).* PLoS One, 2014. **9**(7): p. e103508.  6. Parsel, S.M., et al., *Gastroesophageal and Laryngopharyngeal Reflux Associated With Laryngeal Malignancy: A Systematic Review and Meta-analysis.* Clin Gastroenterol Hepatol, 2019. **17**(7): p. 1253-1264.e5.  7. Eells, A.C., et al., *Gastroesophageal reflux disease and head and neck cancers: A systematic review and meta-analysis.* Am J Otolaryngol, 2020. **41**(6): p. 102653.  8. Saha, B., et al., *Prevalence of Barrett's Esophagus and Esophageal Adenocarcinoma With and Without Gastroesophageal Reflux: A Systematic Review and Meta-analysis.* Clin Gastroenterol Hepatol, 2024. **22**(7): p. 1381-1394.e7.  9. Wang, X., et al., *Association of gastroesophageal reflux disease with the incidence of pulmonary disease.* Front Cell Dev Biol, 2025. **13**: p. 1552126.  10. Rezaii, J., et al., *Association between Helicobacter pylori infection and laryngo-hypopharyngeal carcinoma: a case-control study and review of the literature.* Head Neck, 2008. **30**(12): p. 1624-7.  11. Shivappa, N., J.R. Hébert, and B. Rashidkhani, *Dietary Inflammatory Index and Risk of Esophageal Squamous Cell Cancer in a Case-Control Study from Iran.* Nutr Cancer, 2015. **67**(8): p. 1253-9.  12. Sonnenberg, A., K.O. Turner, and R.M. Genta, *Increased Risk for Colon Polyps in Patients with Reflux Disease.* Dig Dis Sci, 2018. **63**(1): p. 228-233.  13. Menon, S., P. Nightingale, and N. Trudgill, *Chronic Obstructive Pulmonary Disease and the Risk of Esophagitis, Barrett's Esophagus, and Esophageal Adenocarcinoma: A Primary Care Case-Control Study.* J Clin Gastroenterol, 2019. **53**(10): p. e451-e455.  14. Yanes, M., et al., *Antireflux surgery and risk of lung cancer by histological type in a multinational cohort study.* Eur J Cancer, 2020. **138**: p. 80-88.  15. Zhang, X., et al., *Lifestyle Risk Factors, Quality of Life, and Intervention Preferences of Barrett's Esophagus Patients: A Prospective Cohort Study.* Glob Adv Health Med, 2021. **10**: p. 21649561211001346.  16. Andalib, A., et al., *Esophageal cancer after sleeve gastrectomy: a population-based comparative cohort study.* Surg Obes Relat Dis, 2021. **17**(5): p. 879-887.  17. Deissova, T., et al., *Lack of Association between Epidermal Growth Factor or Its Receptor and Reflux Esophagitis, Barrett's Esophagus, and Esophageal Adenocarcinoma: A Case-Control Study.* Dis Markers, 2022. **2022**: p. 8790748.  18. Yanes, M., et al., *Laryngeal and Pharyngeal Squamous Cell Carcinoma After Antireflux Surgery in the 5 Nordic Countries.* Ann Surg, 2022. **276**(2): p. e79-e85.  19. Holmberg, D., et al., *Incidence and Mortality in Upper Gastrointestinal Cancer After Negative Endoscopy for Gastroesophageal Reflux Disease.* Gastroenterology, 2022. **162**(2): p. 431-438.e4.  20. Zhang, X., et al., *Association of educational attainment with esophageal cancer, Barrett's esophagus, and gastroesophageal reflux disease, and the mediating role of modifiable risk factors: A Mendelian randomization study.* Front Public Health, 2023. **11**: p. 1022367.  21. Åkerström, J.H., et al., *Antireflux Surgery Versus Antireflux Medication and Risk of Esophageal Adenocarcinoma in Patients With Barrett's Esophagus.* Gastroenterology, 2024. **166**(1): p. 132-138.e3.  22. Chandar, A.K., et al., *Patients With Esophageal Adenocarcinoma With Prior Gastroesophageal Reflux Disease Symptoms Are Similar to Those Without Gastroesophageal Reflux Disease: A Cross-Sectional Study.* Am J Gastroenterol, 2024. **119**(5): p. 823-829.  23. Byrne, C.J., et al., *Long-term risk factors for developing Barrett's oesophagus in patients with gastro-oesophageal reflux disease: a longitudinal cohort study.* BMJ Open Gastroenterol, 2024. **11**(1).  24. Ku, P.K.M., et al., *The prevalence of gastroesophageal reflux disease and laryngopharyngeal reflux in patients with dysphagia after radiotherapy for nasopharyngeal carcinoma.* Head Neck, 2024. **46**(7): p. 1637-1659.  25. Chiou, T.W., et al., *The incidence of esophageal second primary cancer in head and neck cancer patients.* Medicine (Baltimore), 2025. **104**(17): p. e42181.  26. Veziant, J., et al., *Obesity, sleeve gastrectomy and gastro-esophageal reflux disease.* J Visc Surg, 2023. **160**(2s): p. S47-s54.  27. Wang, S., et al., *Causal analysis of gastroesophageal reflux disease and esophageal cancer.* Medicine (Baltimore), 2024. **103**(11): p. e37433.  28. Edhi, A., et al., *Helicobacter pylori infection does not influence the progression from gastroesophageal reflux disease to Barrett's esophagus to esophageal adenocarcinoma.* Minerva Gastroenterol (Torino), 2024. **70**(4): p. 454-462.  29. Langevin, S.M., et al., *Gastric reflux is an independent risk factor for laryngopharyngeal carcinoma.* Cancer Epidemiol Biomarkers Prev, 2013. **22**(6): p. 1061-8.  30. Sereg-Bahar, M., A. Jerin, and I. Hocevar-Boltezar, *Higher levels of total pepsin and bile acids in the saliva as a possible risk factor for early laryngeal cancer.* Radiol Oncol, 2015. **49**(1): p. 59-64. | | | |

**
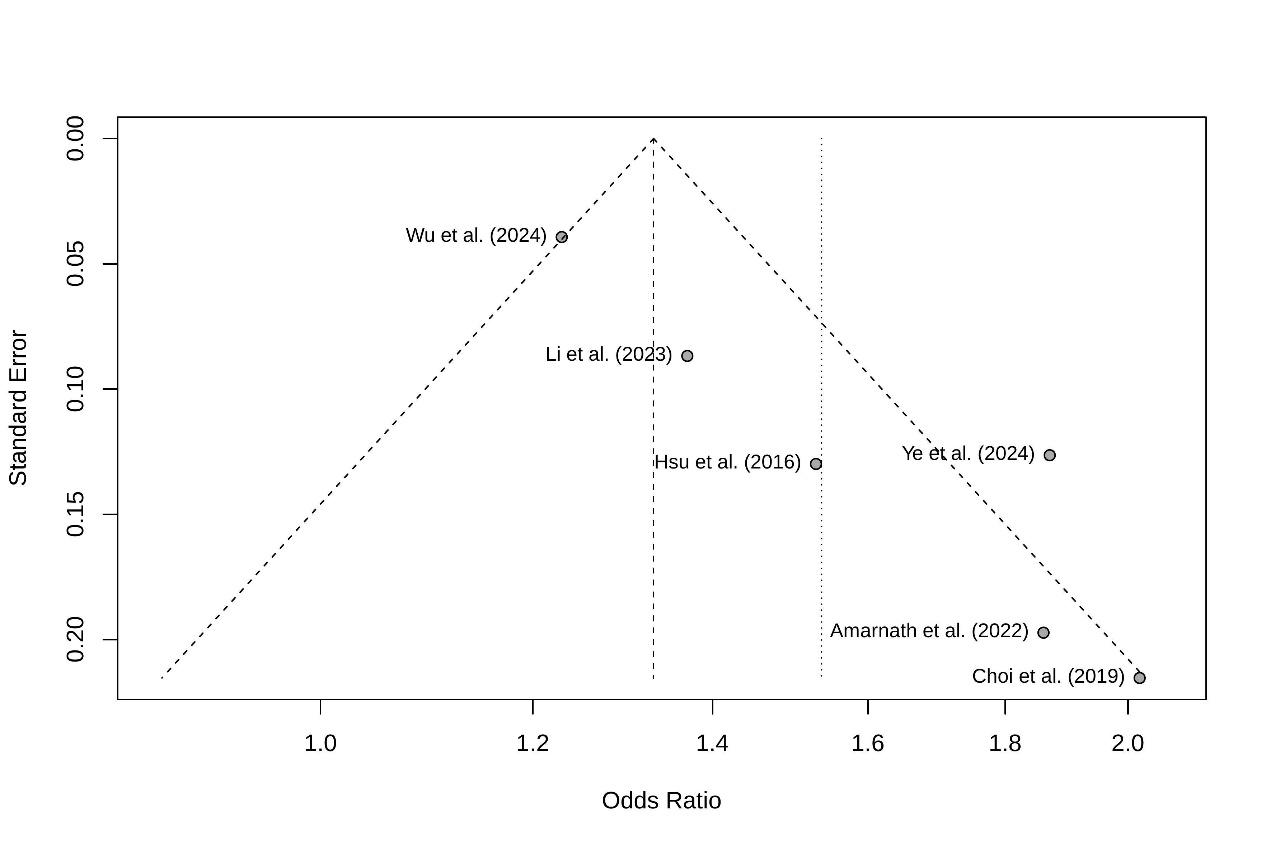
**

**Figure S1. Funnel plot of GERD and the risk of lung cancer**


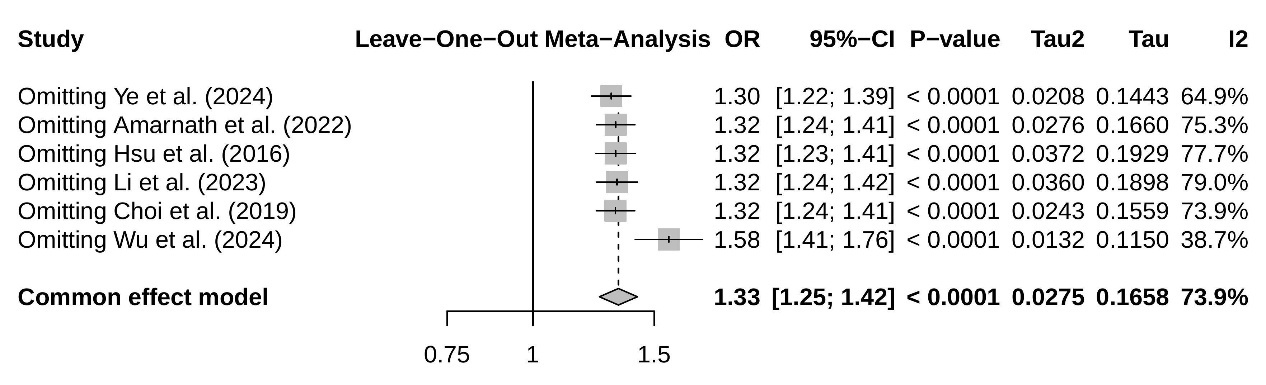


**Figure S2. Sensitivity analysis of GERD and the risk of lung cancer**


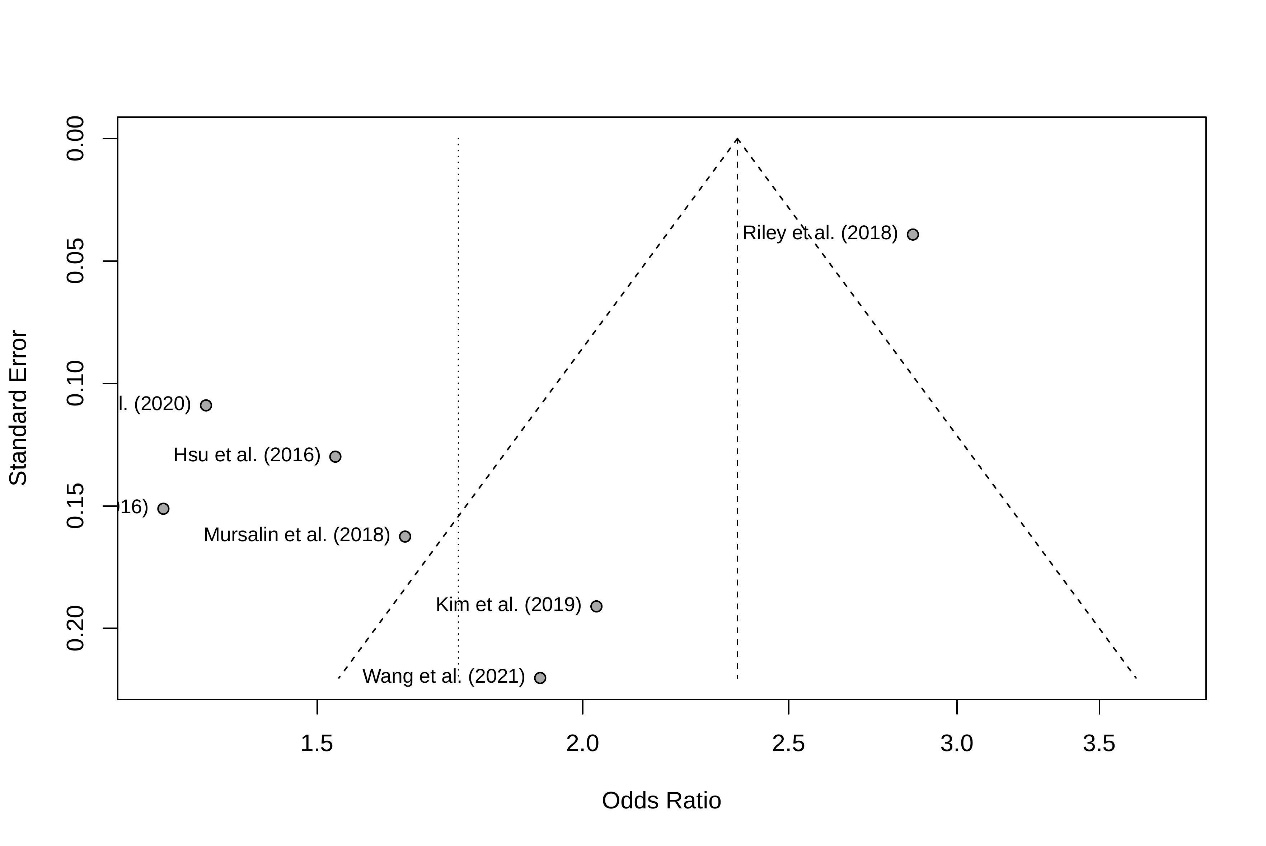


**Figure S3. Funnel plot of GERD and the risk of laryngeal cancer**


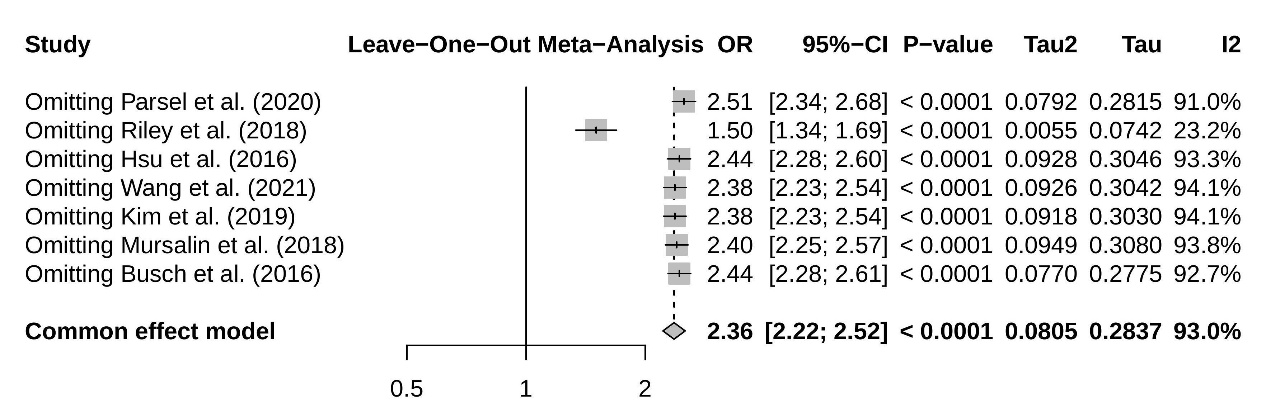


**Figure S4. Sensitivity analysis of GERD and the risk of laryngeal cancer**


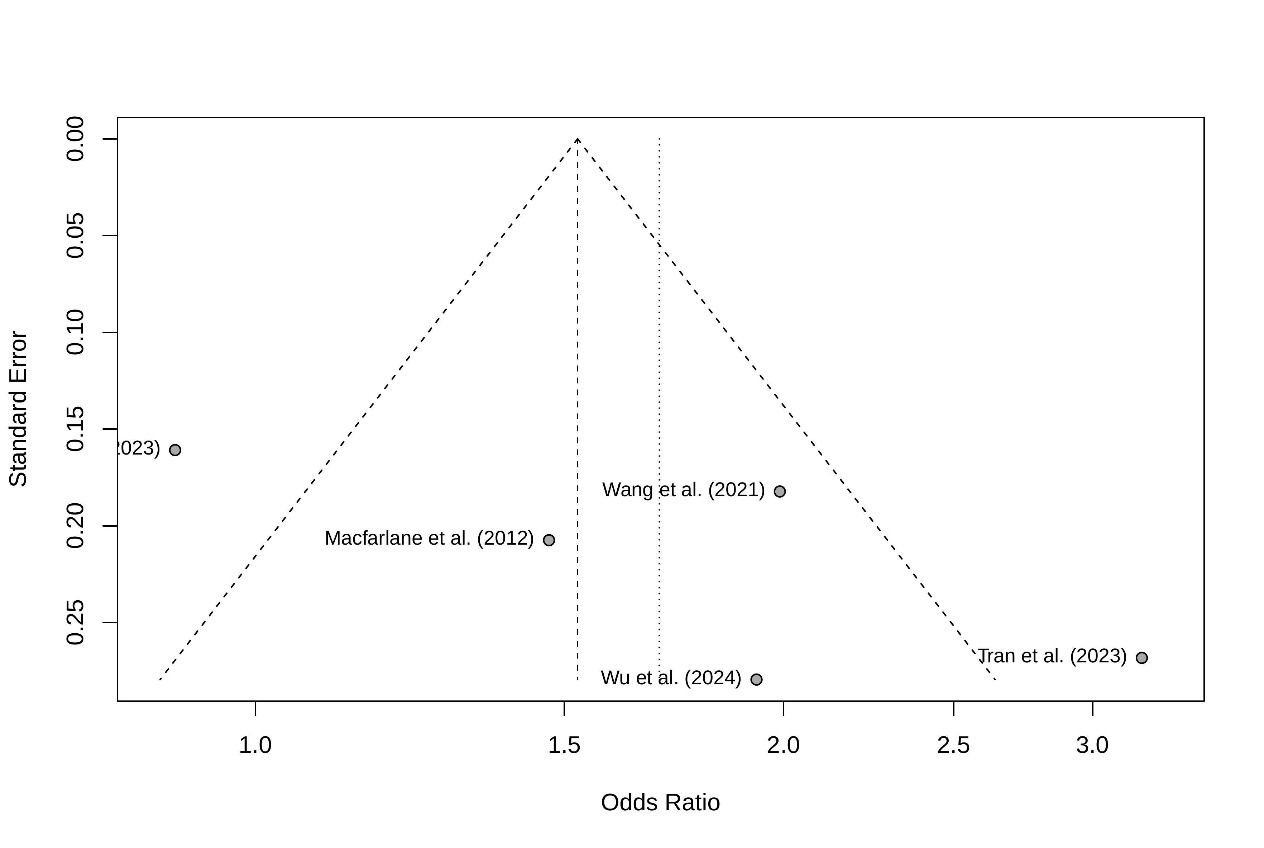


**Figure S5. Funnel plot of GERD and the risk of esophageal cancer**


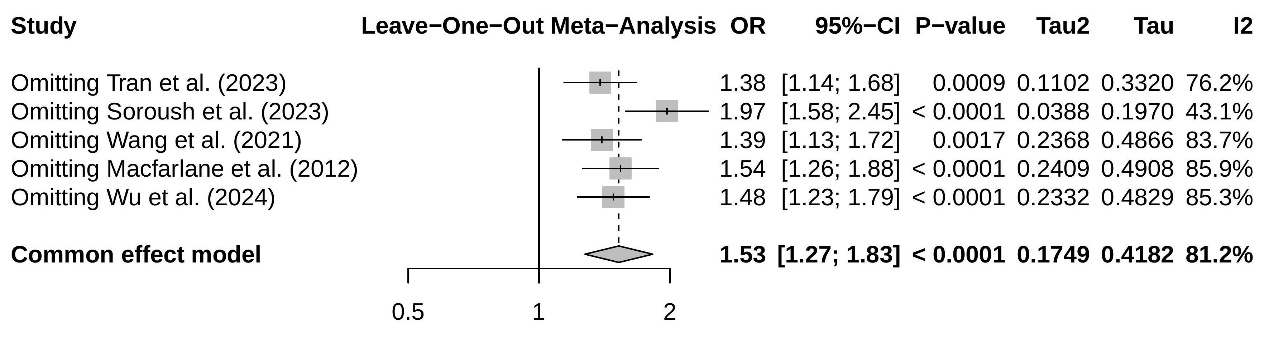


**Figure S6. Sensitivity analysis of GERD and the risk of esophageal cancer**


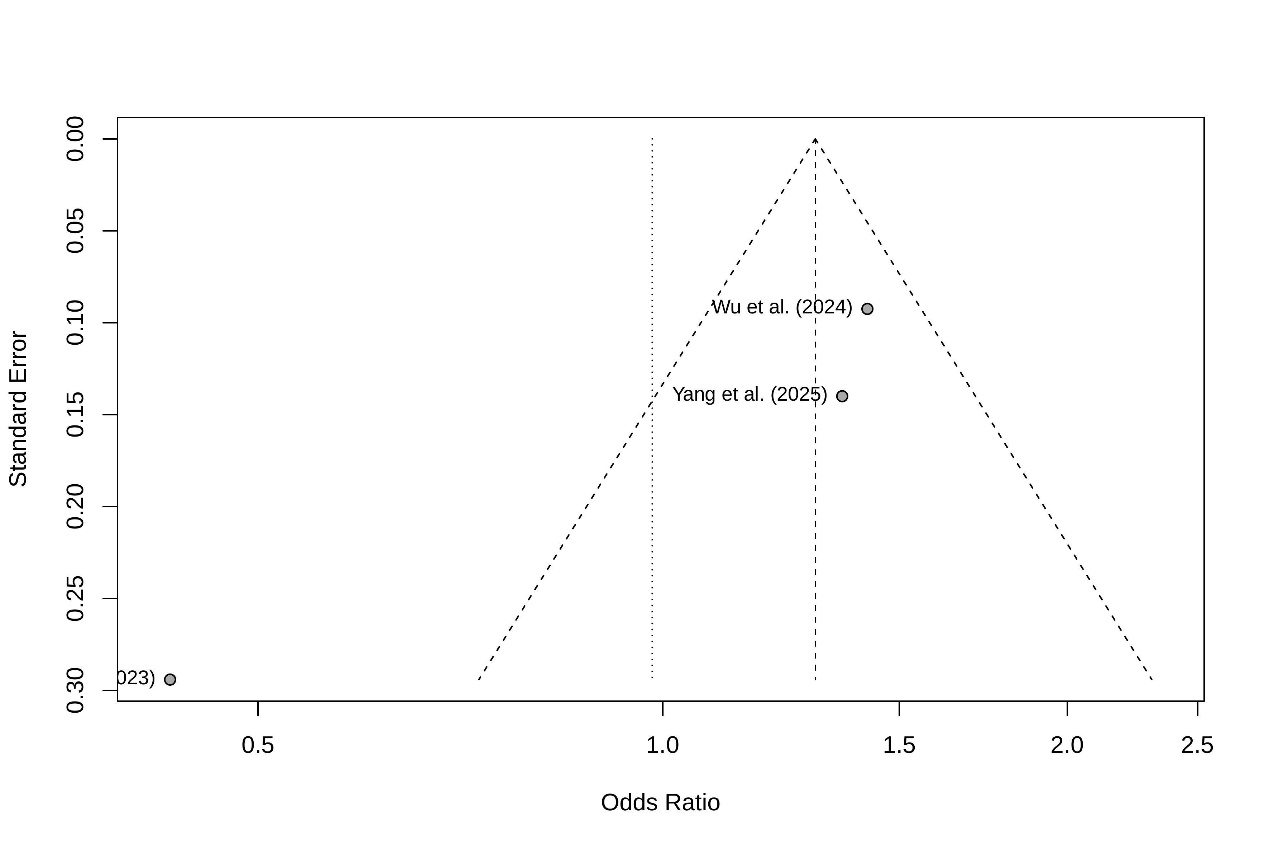


**Figure S7. Funnel plot of GERD and the risk of pancreatic cancer**


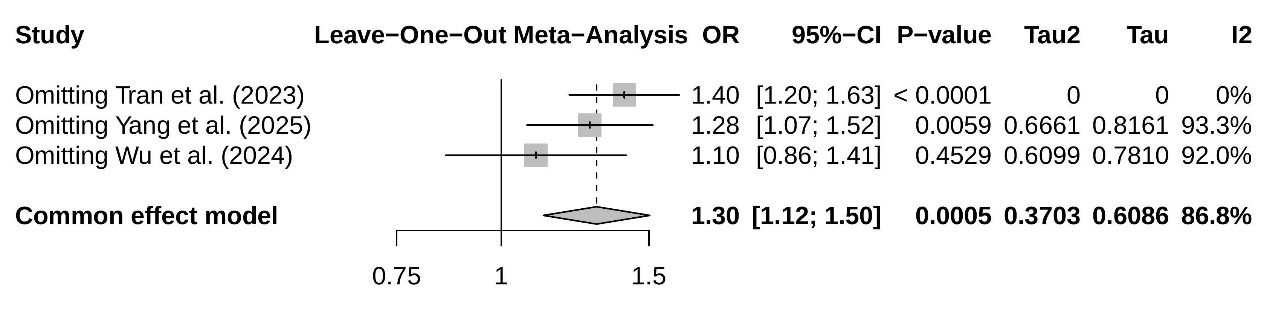


**Figure S8. Sensitivity analysis of GERD and the risk of pancreatic cancer**


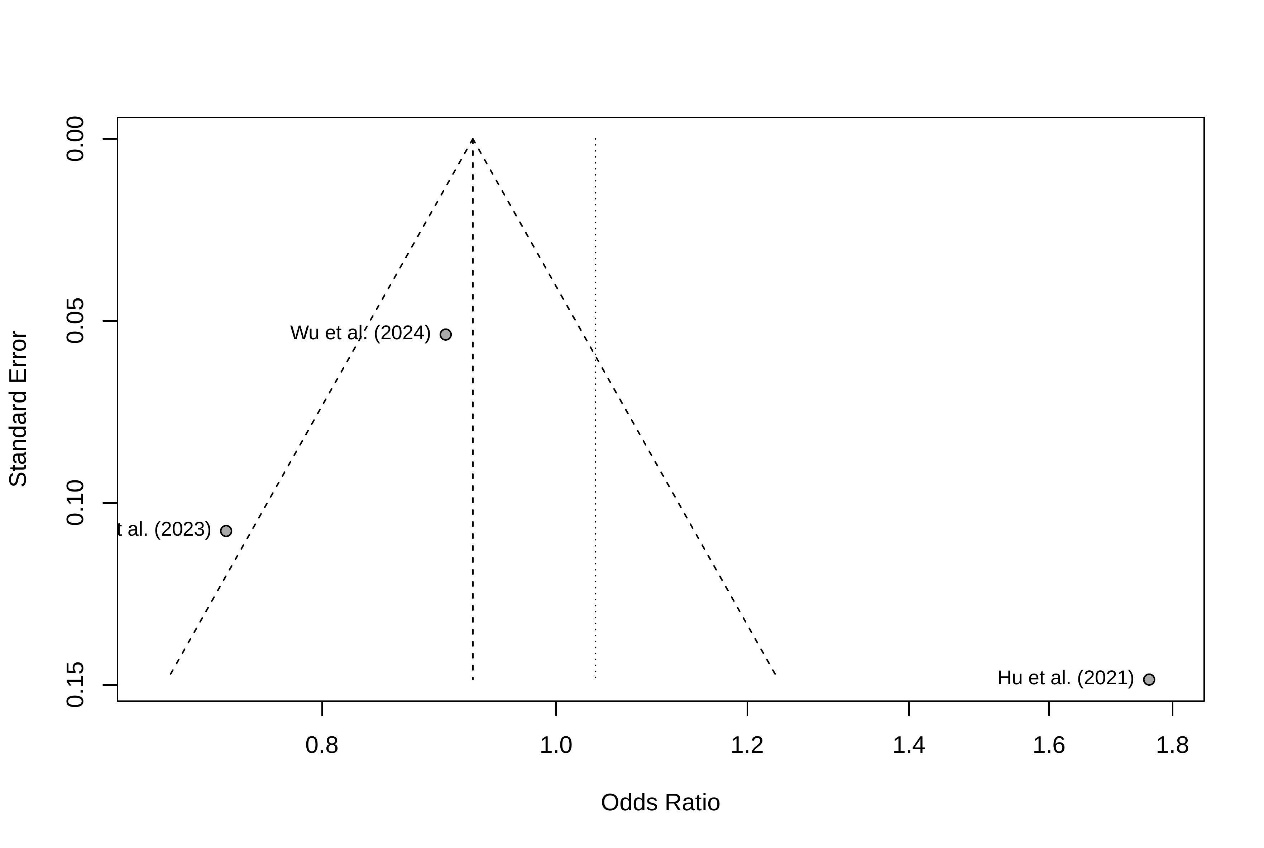


**Figure S9. Funnel plot of GERD and the risk of colorectal cancer**


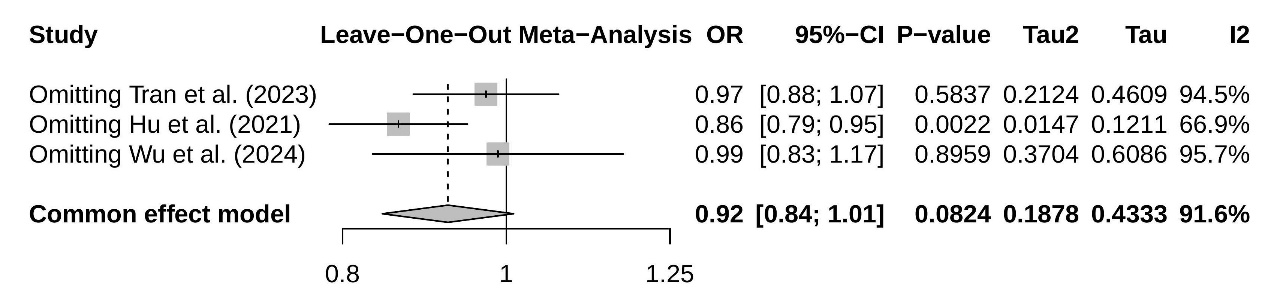


**Figure S10. Sensitivity analysis of GERD and the risk of colorectal cancer**
